# Living with osteoarthritis is a balancing act: an exploration of patients’ beliefs about knee pain

**DOI:** 10.1186/s41927-018-0023-x

**Published:** 2018-06-12

**Authors:** Ben Darlow, Melanie Brown, Bronwyn Thompson, Ben Hudson, Rebecca Grainger, Eileen McKinlay, J. Haxby Abbott

**Affiliations:** 1 Department of Primary Health Care and General Practice, University of Otago - Wellington, Wellington, New Zealand; 2 Department Orthopaedic Surgery & Musculoskeletal Medicine, University of Otago - Christchurch, Christchurch, New Zealand; 3 Department of General Practice, University of Otago - Christchurch, Christchurch, New Zealand; 4 Department of Medicine, University of Otago - Wellington, Wellington, New Zealand; 5 Department of Surgical Sciences, University of Otago, Dunedin, New Zealand

**Keywords:** Osteoarthritis, Knee, Patient perceptions, Health knowledge, attitudes, practice, Qualitative research

## Abstract

**Background:**

This study aimed to explore the beliefs of people with knee osteoarthritis (OA) about the disease, and how these beliefs had formed and what impact these beliefs had on activity participation, health behaviour, and self-management.

**Methods:**

Semi-structured interviews were conducted with 13 people with knee OA recruited from general practices, community physiotherapy clinics, and public advertisements in two provinces of New Zealand. Data were analysed using Interpretive Description.

**Results:**

Two key themes emerged. 1) *Knowledge: certainty and uncertainty* described participants’ strong beliefs about anatomical changes in their knee. Participants’ beliefs in a biomechanical model of progressive joint degradation often appeared to originate within clinical encounters and from literal interpretation of the term ‘wear and tear’. These beliefs led to uncertainty regarding interpretation of daily symptoms and participants’ ability to influence the rate of decline and certainty that joint replacement surgery represented the only effective solution to fix the damaged knee. 2) *Living with OA* described broader perspectives of living with OA and the perceived need to balance competing values and risks when making decisions about activity participation, medication, attentional focus, accessing care, and making the most of today without sabotaging tomorrow. Misunderstandings about knee OA negatively impacted on activity participation, health behaviours, and self-management decisions.

**Conclusion:**

Biomechanical models of OA reduced participant exploration of management options and underpinned a perceived need to balance competing values. Improved information provision to people with knee OA could help guide positive health behaviour and self-management decisions and ensure these decisions are grounded in current evidence.

**Electronic supplementary material:**

The online version of this article (10.1186/s41927-018-0023-x) contains supplementary material, which is available to authorized users.

## Background

Osteoarthritis (OA) is a common condition that causes considerable disability and high levels of health expenditure [1, 2]. Knee OA accounts for over 80% of the total OA disease burden [3] and its prevalence is rapidly increasing [4]. This will have considerable social and economic consequences, particularly as people with OA are twice as likely to be absent from work or retire early due to ill-health [5, 6].

People’s beliefs about knee OA have an important impact on their lived experience of the disease, influencing activity levels, social and leisure participation, and emotional wellbeing [7, 8]. Beliefs about knee OA aetiology have been explored in a number of qualitative studies, indicating that many people consider OA to be an inevitable part of ageing that is influenced by wear and tear due to joint use and obesity [9–12]. There is a commonly held mechanical view of OA that focuses on loss of cartilage and bone abutting directly on bone [10, 13].

Physical inactivity may predispose to knee OA [4, 14] and physical activity is a key tenet of OA management recommendations [15]. Beliefs about physical activity among people with knee OA have been explored in the context of general physical activity [16] and adherence to exercise-based OA self-management programmes [9, 17]. Beliefs that OA is caused by wear and tear result in worry that weight bearing exercise will exacerbate joint damage [17] and these concerns may cause people to reduce activity levels or avoid activities [16].

There is currently inadequate understanding of how people’s beliefs about knee OA are informed. Given the discordance between evidence-based physical activity recommendations for knee OA and consumer beliefs about OA aetiology and the role of activity, it is important to address the gap in the literature regarding *how* people’s beliefs about knee OA and activity are formed and *what specific impact* these beliefs have on activity participation and self-management. Improved understanding of how beliefs are formed and factors that influence this process may enable clinicians to positively influence beliefs about knee OA and improve people’s experience of living with this condition.

This qualitative study aimed to explore the beliefs of New Zealanders with knee OA about the disease, and in particular, how these beliefs had formed and what specific impact these beliefs had on activity participation, health behaviour, and self-management.

## Methods

This study adhered to the Consolidated criteria for reporting qualitative studies (COREQ; Additional file 1).

### Research design

Qualitative data were gathered and analysed using Interpretive Description [18, 19]. This methodology aims to inform clinical understanding by identifying themes and patterns within participant perspectives [18, 19]. The study complied with the Declaration of Helsinki. The University of Otago Human Ethics Committee (Health) (H15/081) approved the study and participants gave written informed consent.

### Recruitment

Participants were recruited in two provinces of New Zealand from general practices and community physiotherapy clinics, and from advertisements to Arthritis New Zealand members and in public areas such as libraries, swimming pools, and supermarkets.

Participants were included in the study if they had been told by a health care professional that they had knee OA. Participants were excluded if they had received a total knee replacement or could not speak English. There was no age limitation. Purposive sampling maximised the range of viewpoints in terms of gender, age, cultural backgrounds, disease severity, and level of functional limitation [20].

### Data collection

Participants were interviewed by experienced qualitative researchers (MB or BT) in a location of their choice; for most, this was the participant’s home. Participants were unknown to interviewers prior to recruitment and were asked to speak to interviewers as lay people. Interviews were conducted in person; face-to-face or by web-based video-conferencing. Participants were able to have a support person present, but all chose to be interviewed alone. A semi-structured interview schedule was developed using questions framed around research aims, but kept flexible to allow participants to focus on what they deemed important (Table 1). Interviews consisted of open-ended questions to elicit the participants’ views on their experiences and perceptions of knee OA, including activity. Afterward, participants completed a demographic questionnaire including self-reported duration of knee pain and clinicians consulted, the Oxford Knee Scale [21] to indicate functional limitation, and the Pain Self-Efficacy Questionnaire [22] to indicate confidence in performing activities despite pain. Interviews were audio-recorded and transcribed verbatim; field notes were also kept.

**Table 1:** Semi-structured interview guide

1. Please tell me about your knee problem from the beginning?	
2. How would you describe your pain?	
3. What do you think is happening in or around your knee	
4. Can you tell me about the things that affect your knee problem?	
5. Is there anything that concerns you about your knee problem?	
6. How have you found out about what is wrong with your knee?	
7. What do you think is the best way to manage your knee problem?	
8. What are your expectations for the future with regards to your knee problem?	

### Data analysis

Recruitment, data collection, and data analysis occurred concurrently to enable collected data to inform subsequent interviews and to cease recruitment once theme saturation was achieved. Data were managed using NVivo 11 software (QSR International Pty Ltd).

Initial transcript coding was undertaken independently by MB and BT on a line-by-line basis using ‘open coding’ to allow multiple codes to be applied to single segments of data. These researchers subsequently discussed and agreed on codes and categories within each transcript. The relationships between and within categories emerging from this process were explored with increasingly higher levels of conceptualisation. Negative case analysis was used to broaden understandings and challenge initial interpretations of the data [20]. Transcripts and coding were crosschecked by another researcher (BD), with all disagreements resolved through regular discussions. Theme documentation was checked and discussed with other authors (BH/RG/JHA/EM) following eight interviews and again following thirteen interviews to further develop the emerging analysis, ensure themes represented participants’ reported experiences and views, and test assumptions related to theme saturation. Consistent with Interpretive Description, participants did not review transcripts or validate findings [19] and there were no repeat interviews.

### Research team

The research team consisted of academics and clinicians with backgrounds in family medicine (BH), health coaching (MB), nursing (EM), occupational therapy (BT), physiotherapy (BD/JHA), and rheumatology (RG). Several researchers (BD/MB/BT/JHA/RG/EM) had experience with qualitative research in musculoskeletal pain.

## Results

Thirteen participants were interviewed (Table 2). Ten further eligible respondents were not interviewed because their characteristics were similar to previous participants (*n* = 7), they changed their mind (*n* = 1), or were unable to schedule time for the interview (*n* = 2). Interviews lasted 60 to 90 min. Data saturation was achieved after eight interviews. Five further participants were purposively recruited and interviewed, but no further themes or significant variations on existing themes emerged.

**Table 2:** Participant characteristics

Pseudonym Gender	Age	Ethnicity	Occupational category	Knee pain duration	Clinical consultation for knee pain	OKS^a^	PSEQ^b^
Geoff, Male	60–64	NZE	Community & personal service worker	14–16 years	Family doctor	26	35
James, Male	70–74	NZE	Retired professional	4–6 years	Family doctor, orthopaedic surgeon,	18	17
Anne, Female	60–64	NZE	Clerical & administrative worker	10–12 years	Family doctor, orthopaedic surgeon	25	49
George, Male	80–84	NZE	Retired professional	8–10 years	Orthopaedic surgeon	25	54
John, Male	65–69	NZE	Professional	4–6 years	Family doctor, orthopaedic surgeon, physiotherapist	42	17
Iosefo, Male	70–74	Samoan	Labourer	4–6 years	Family doctor	22	60
Tui, Female	60–64	Māori	Retired professional	6–12 months	Family doctor, nurse, orthopaedic surgeon,	57	32
Linda, Female	50–54	Danish	Professional	20+ years	Family doctor, orthopaedic surgeon	34	16
Karen, Female	60–64	NZE	Clerical & administrative worker	18–20 years	Family doctor, orthopaedic surgeon, physiotherapist	24	52
Susan,Female	60–64	NZE	Professional	1–2 years	Bowen therapist, family doctor, Reiki practitioner	32	40
Mary, Female	70–74	NZE	Retired	14–16 years	Family doctor, orthopaedic surgeon, physiotherapist	36	35
Brenda, Female	55–59	NZE	Professional	0–6 months	Acupuncturist, family doctor, homeopath, homeopathic chemist, naturopath, physiotherapist	40	27
William, Male	60–64	NZE	Community & personal service worker	4–6 years	Acupuncturist; chiropractor, family doctor; orthopaedic surgeon, osteopath,, physiotherapist	22	56

*NZE* New Zealand European, *OKS* Oxford Knee Scale, *PSEQ* Pain Self Efficacy Questionnaire

^a^Scored on a range from 12 to 60 with higher scores indicating greater functional limitation

^b^Scored on a range from 0 to 60 with higher scores indicating greater confidence in performing activities despite pain

Two overarching themes emerged from the data;, *Knowledge: certainty and uncertainty* and *Living with osteoarthritis*. Findings are presented with illustrative extracts from participants’ interviews; additional quotes are in Additional files 2 and 3. An additional theme emerged around *Health System Support*; as this large theme was conceptually distinct and unrelated to the primary aims of this study, it will be presented in a future publication.

### Knowledge: certainty and uncertainty

This theme described participants’ beliefs about OA and how it should be managed, describing how these beliefs have been formed and influenced as well as the impact of these beliefs.

#### Structural model of progressive degeneration

Participants used descriptive language and imagery to express their strong beliefs about anatomical and pathological changes in their knees. The phrases ‘wear and tear’, ‘bone-on-bone’, and ‘missing cartilage’ were used frequently to explain their understanding of OA:

*“It doesn’t take a rocket scientist to work out that [it’s bone-on-bone]. If the fluid between the ball-bearing and the thing has all gone, you know, it’s like a car situation.”*

*–Tui*

For many, certainty about this biomechanical model of structural deterioration led to a matter-of-fact attitude, and often a lack of curiosity about seeking information or exploring management options:

*“It’s sort of like a pound of butter. That’s what it is! It’s butter. Arthritis is arthritis.”*

*–George*

Despite the sense of certainty around the biomechanical model, participants often had no explanations for the variability of symptoms, the speed of potential degradation, or the best ways to optimise function and slow deterioration. This model of progressive deterioration was in conflict with some participants’ experiences of stable or improving symptoms. Participants appeared not to recognise this discordance.

Participants saw ‘wear and tear’ as synonymous with OA and interpreted the concept literally. Consequently, participants felt that they needed to protect their joint to prevent further wear and tear. These concepts were often reported as originating with, or being reinforced by, health professionals:

*“They always say same thing: wear and tear, you know, you’re getting older.”*

*–Iosefo*

Many participants had been shown X-rays that provided graphic evidence of the loss of space between bone ends. Participants expressed shock at seeing these changes; several explained that the X-rays led them to believe that they needed to protect their knee from further damage. These concepts were reinforced by what participants saw, heard, and felt from their knee (such as a bowed appearance, grinding, or knocking):

*“It’s really obvious I have no cushioning in that knee.”*

*–John*

#### Approaches to osteoarthritis management

The strongly-held model of ongoing structural deterioration led to participants using strategies such as: avoiding, reducing, or pacing activities to limit wear and tear; participating in activities they considered not to cause joint impact; and taking natural supplements to lubricate the joints (e.g. fish oil) or feed the cartilage (e.g. glucosamine or gelatine). Two participants (‘Linda’ and ‘William’) attributed their successes in managing OA to specific weight-loss management or strength-based exercise regimes.

Pain or stiffness guided activity participation or avoidance, but participants were often uncertain about these choices. Some participants interpreted pain that lasted after they stopped exercise as a sign of further damage, whereas pain that abated was a reminder to be careful. Participants’ understandings of helpful and unhelpful strategies were strongly influenced by their structural model of what was happening to, and beliefs about what might be safe or good for, the knee:

*“[Biking] there’s no load on your knees… it’s keeping you in motion, keeping you active, and it’s not stress or anything on your knees.”*

*–Anne*

Participants often spoke about stages of management related to the degree of joint degeneration. These included things they had done in the past and things they may try in future. Participants planned to continue with their current strategies until these were no longer effective, indicating progression to another stage. Differing stages helped explain why certain remedies might work for some people but not others:

*“All that sort of stuff [like glucosamine] is supposed to help your cartilage and protect it. But once it’s not there, it’s not going to make more of it … once it’s gone it’s gone.”*

*–Karen*

Although participants expressed hope that they may be able to maintain the status quo or slow the rate of deterioration, they were generally resigned to progressive degradation over which they had little control. The inevitability of further deterioration was supported by the beliefs that OA is part of ageing and the joint is worn away by movement.

*“I don’t think it would improve. It may stay the same, but I would expect it to get worse… you can’t change osteoarthritis.”*

*–Karen*

Participants anticipated increasing pain and activity limitation, which would reduce their quality of life:

*“It worries me that one day I won’t be able to do the things I can do today.”*

*–Linda*

Participants believed joint replacement surgery is inevitable and represents the only effective solution to fix OA (albeit a temporary fix, as they expected the joint replacement would also wear out). This belief was expressed most participants including those with early-stage OA and those who expressed positive expectations around managing day-to-day.

### Living with osteoarthritis

This theme described broader perspectives of living with OA and the decisions and trade-offs that participants made based on their beliefs about OA.

#### The big picture

When discussing the meaning of symptoms and symptom fluctuation participants did not usually talk about structural changes within their knee. Rather, they talked about how pain and symptoms limited activity and day-to-day life, and OA’s broader effects on, and interactions with, mood, wellbeing, and sleep.

Participants often downplayed OA and used minimising language to discuss the condition and its effects. Regardless of age of onset, participants saw OA as part of getting older. Participants perceived OA was not as serious as other health conditions, such as cancer, and a topic that would bore their peers or health professionals. Some participants explicitly downplayed the condition to reduce OA’s place in their lives, or avoid being perceived as a moaner or identifying as ‘someone with arthritis’.

*“I decided I don’t want this to define me, I’m much more than my knee.”*

*–Susan*

Similarly, many perceived OA was downplayed by clinicians; some commented that this was harmful:

*“I’ve made a decision not to use that [term ‘wear and tear’]… the implication is that it’s not unusual and everybody gets it and, you know, it’s not something we need to take any notice of.”*

*–Brenda*

Participants knew that they could not keep playing it down forever. They anticipated a time when OA would affect more than day-to-day activities and begin to affect their core identity and sense of self. Loss of satisfaction or identity were key indicators that it was time for surgery.

Participants discussed benefits of exercise for general physical and mental health and for managing comorbidities. Some participants reported exercising despite concerns about further wear and tear:

*“I’m prepared to face up with a bit of further degeneration in my right knee if everything else benefits.”*

*–George*

#### Living with osteoarthritis is a balancing act

**Fig. 1:**
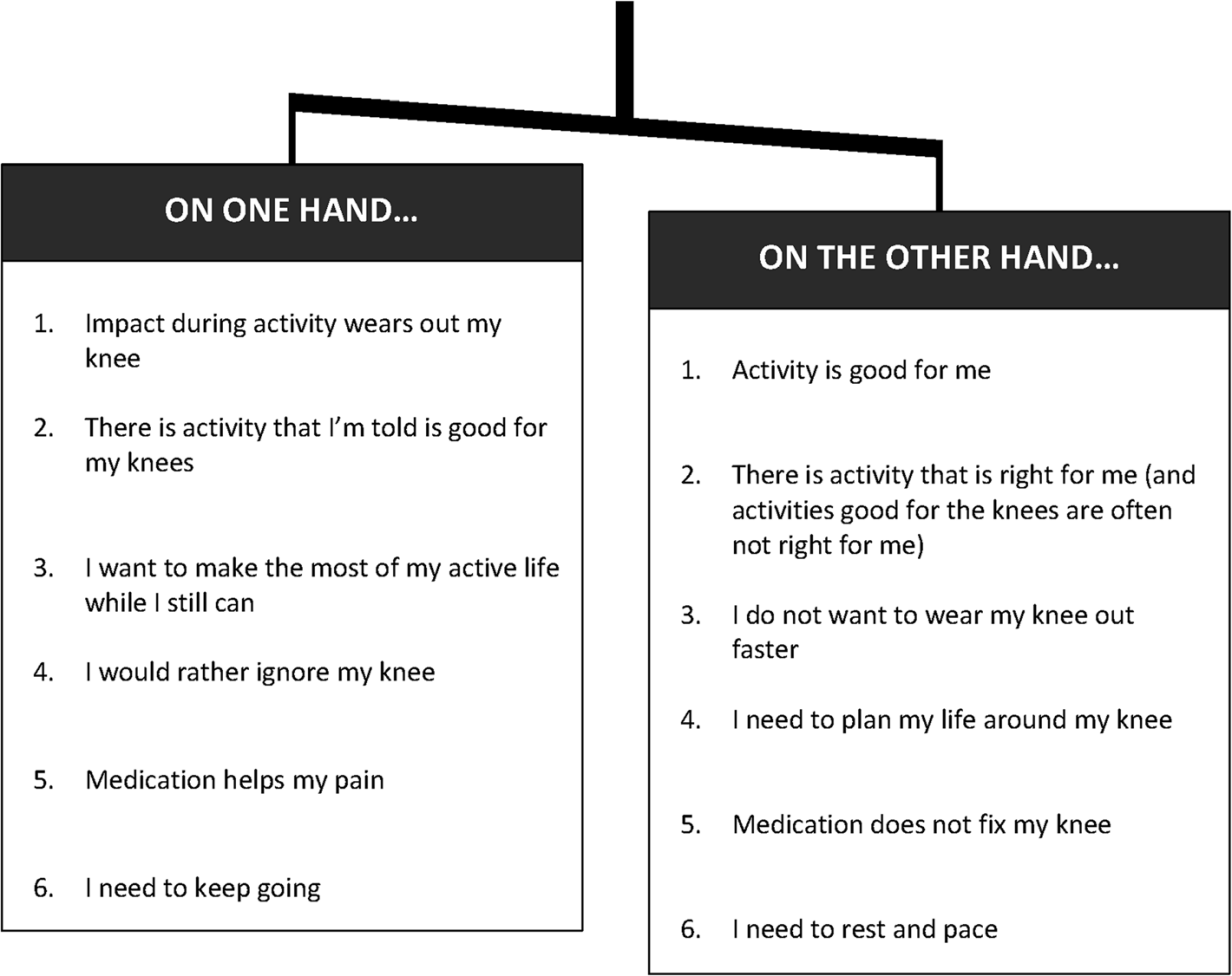
The balance between competing values and risks described by participants living with knee osteoarthritis

Participants saw living with OA as a ‘balancing act’. Participants’ understandings of OA and expectations of future decline, combined with uncertainty about the meaning of fluctuating symptoms and effects of exercise or movement, led to balancing competing values and risks (Fig. 1). On the one hand, participants identified benefits of activity for their knee and general health, but on the other hand they were concerned about increasing pain or further joint degradation:

*“Am I strengthening it or am I sort of destroying the cartilage? I don’t know.”*

*–William*

Participants considered the safety of an exercise for the knee when weighing benefits against costs or risks. However, ‘safe’ activities were not always activities participants liked, felt comfortable with, or were able to do. There was also tension between the notion of the knee needing rest, pacing, and protection versus the need to keep moving, to keep one’s identity, and to get on with things.

*“It’s the balance between activity and rest for the joint… I’m probably wearing it more, but weighing that up against not doing anything then everything else will fall apart.”*

*–Susan*

Participants spoke about the importance of putting OA out of their minds and not letting it define their identity but simultaneously balancing this with the need to plan and choose activities.

*“It’s not always at the front of my mind, but it’s probably always in the back of my mind… because it has to be.”*

*–Karen*

Expectation of progressive decline meant participants wanted to be active while still able, but equally they were concerned that activity would accelerate their joint degradation:

*“I’ve got a window of time to do all these things in. But then, at the same time, I’ve got to do this in a way that doesn’t impact that window of time, make it shorter than it otherwise would be. So it’s really, yeah, finding that balance.”*

*–Linda*

Many participants mentioned the cost-benefit payoff between medication and side effects, especially as analgesics were not addressing the perceived inevitable degeneration:

*“All it’s really doing is taking the pain away a little bit. But the joint continues to deteriorate, the pain gets worse.”*

*–James*

Many participants wanted to delay surgery as long as possible due to concerns about surgery, recovery times, and uncertainty about how long the new joint would last. However, they were also concerned about the impact of increasing age or disability on joint replacement outcomes.

## Discussion

This study explored beliefs about the OA disease process and impact in people with knee OA. In particular, and not fully explored in previous literature, it also explored belief formation and influence on decisions about activity participation, health behaviours, and self-management. Irrespective of duration or severity of knee symptoms, all participants viewed OA as progressive joint degradation due to wear and tear and ageing. Participants’ biomechanical explanation of symptoms and expectations of inevitable decline appeared to be derived from, or perpetuated by, clinicians’ language and explanations. Despite the limited correlation between X-ray findings and symptoms or disability [23], participants considered that their symptoms directly reflected their joint surface condition as seen on X-ray. Previous studies have indicated that clinicians may trivialise or minimise OA and associate it with old age and these findings were supported by the current study [9, 11, 24, 25]. This may influence a fatalistic attitude amongst people with knee OA and pessimism about engaging in care [9–11].

Participants preferred to discuss the impact of knee OA on their daily lives rather than explain their understanding of the biological mechanisms involved, which they saw as straightforward. Consistent with findings from people with rheumatoid arthritis, participants sought a balance between managing OA and living their daily lives, and matched management strategies to their perceived stage of disease [26]. Pouli et al. [10] found that people with knee OA weigh the pain relieving benefits of medication against the negative side effects or risk of dependence. The current study expands understanding of the range of factors people with knee OA balance in their daily lives. These include balancing benefits of physical activity against risks, ‘safe’ activities against activities they enjoyed, putting OA out of their mind while also planning lives around it, not being perceived as a moaner while also accessing necessary care, and making the most of their current function without jeopardising the future.

This study confirms that those with knee OA are often cautious of physical activity due to fear of accelerating joint degradation [12, 16, 17], and that some people engage in activity despite concerns or expectations of damage because of perceived benefits related to their general health and well-being [17]. These beliefs conflict with research demonstrating that exercise improves cartilage volumes, is safe for people with OA, and improves pain and function [27–31].

A number of studies have reported ambivalent views about joint replacement surgery in people with knee OA as a result of concerns about the surgery effectiveness, surgical risk, recovery times, and a compromised sense of internal control [10, 11, 24]. These concerns were discussed by participants in the current study, however, they strongly believed that joint replacement surgery was the only way to fix their knee joint and an inevitable part of their clinical journey. Reasons for this discrepancy could be explored with future research. Expectations of inevitable decline and ultimate joint replacement surgery decreased exploration of, and engagement in, strategies to improve joint function and health.

### Strengths and limitations

The qualitative methodology allowed in-depth exploration of participants’ beliefs about knee OA. Transcripts were independently analysed by two researchers to increase rigour and all findings were reviewed and debated by the entire interdisciplinary research team. Participants were recruited from two geographically separate provinces of New Zealand and the sampling frame enabled inclusion of participants with a range of characteristics. There was no age restriction, however, no participants under 50 years of age volunteered to participate. Consequently, this study does not represent the views of younger people with knee OA, however, it does represent the main age group affected. Recruitment continued until no new themes emerged from the data. Saturation was achieved after eight interviews, demonstrating strong commonalities in language and beliefs despite differences in background, disease severity, and functional limitation. This study was not designed to explore differences between subgroups with different characteristics (e.g. length of symptoms or disease severity), however, beliefs and conceptual frameworks were surprisingly consistent. Information provided to participants may have been different from what they reported, however, the use of ‘wear and tear’ and minimisation of OA by clinicians has been directly observed in consultations [25]. Although these findings are consistent with those found with other populations [13, 16, 17], caution is advised when applying these findings to other settings. The inclusion of consumers as part of the research team could have added insights to the analysis.

### Implications for clinical practice and future research

People with OA make difficult decisions on a daily basis, but many decisions are premised on inaccurate information or beliefs that are often not addressed, and may even be promulgated, by clinicians. Clinicians’ use of the term ‘wear and tear’ may represent an attempt to present the diagnosis of OA in a less threatening way or an effort to shift focus from the diagnosis of ‘osteoarthritis’ to strategies for managing symptoms and improving function [25, 32]. However, participants saw ‘*wear and tear*’ as being synonymous with ‘*osteoarthritis*’, so it did not reduce the threat associated with diagnosis. Literal interpretation of ‘wear and tear’ established inaccurate biomechanical models that reinforced the perceived need to limit activity to protect the joint and thereby prevented engagement in positive self-management. Minimisation associated with ‘it’s just wear and tear’ may also limit access to appropriate care and reduce the perceived need to engage in proactive self-management and behaviour change.

Information provided to people with OA should focus on living with OA rather than biomedical aspects of the disease [33]. The current study highlights a need to address unhelpful or inaccurate language and beliefs. Participants’ universal adoption of a biomedical model limited activity participation, increased uncertainty, negatively influenced expectations for the future, and forced people with knee OA to make unnecessary decisions and trade-offs. These findings will be used to inform the development of novel information resources. Future research should explore the impact of information resources on modifying patients’ beliefs about knee OA and empowering increased participation in activities and behaviours known to improve pain, function, and experiences of living with OA.

## Conclusions

Participants’ biomechanical models of OA and expectations of inevitable decline were influenced by clinicians’ language and explanations. These beliefs reduced participant exploration of management options and underpinned a perceived need to balance competing values. Improved information provision to people with knee OA could help guide positive health behaviour and self-management decisions and ensure these decisions are grounded in current evidence.

## Additional files

Additional file 1: Consolidated criteria for reporting qualitative studies (COREQ) checklist. (PDF 280 kb)

Additional file 2: Participant data supporting Theme 1 – Knowledge: Certainty and Uncertainty. Additional data to support theme 1. (PDF 490 kb)

Additional file 3: Participant data supporting Theme 2 – Living with osteoarthritis. Additional data to support theme 2. (PDF 485 kb)

## Abbreviation

OA: Osteoarthritis
