# Supplementary material for: Living with osteoarthritis is a balancing act: an exploration of patients’ beliefs about knee pain
Source: BMC Rheumatol. 2018 Jun 12;2:15. doi: 10.1186/s41927-018-0023-x (PMC6390552; doi:10.1186/s41927-018-0023-x)
Supplement: Supplementary file 1 — Consolidated criteria for reporting qualitative studies (COREQ) checklist. (PDF 280 kb) [file 41927_2018_23_MOESM1_ESM.pdf]

Additional file 1. Consolidated criteria for reporting qualitative studies (COREQ): 32-item checklist

No.	Item	Section	Page
1	Interviewer/facilitator	Main Text	4
2	Credentials	Title Page	1
3	Occupation	Title Page	1
4	Gender	Title Page	1
5	Experience and training	Main Text	4
6	Relationship established	Main Text	4
7	Participant knowledge of the interviewer	Main Text	4
8	Interviewer characteristics	Main Text	4
9	Methodological orientation and Theory	Main Text	4
10	Sampling	Main Text	4
11	Method of approach	Main Text	4
12	Sample size	Main Text	6
13	Non-participation	Main Text	6
14	Setting of data collection	Main Text	4
15	Presence of non-participants	Main Text	5
16	Description of sample	Table 2	19
17	Interview guide	Table 1	5
18	Repeat interviews	Main Text	6
19	Audio/visual recording	Main Text	5
20	Field notes	Main Text	5
21	Duration	Main Text	6
22	Data saturation	Main Text	6
23	Transcripts returned	Main Text	6
24	Number of data coders	Main Text	6
25	Description of the coding tree	Main Text	6
26	Derivation of themes	Main Text	6
27	Software	Main Text	5
28	Participant checking	Main Text	6
29	Quotations presented	Main Text	7 - 13
30	Data and findings consistent	Main Text	7 – 13
31	Clarity of major themes	Main Text	7 – 13
32	Clarity of minor themes	Main Text	7 – 13

From: Darlow B, Brown M, Thompson B, Hudson B, Grainger R, McKinlay E, Abbott JH (2018) Living with osteoarthritis is a balancing act: An exploration of patients' beliefs about knee pain.
