# Supplementary material for: Living with osteoarthritis is a balancing act: an exploration of patients’ beliefs about knee pain
Source: BMC Rheumatol. 2018 Jun 12;2:15. doi: 10.1186/s41927-018-0023-x (PMC6390552; doi:10.1186/s41927-018-0023-x)
Supplement: Supplementary file 2 — Participant data supporting Theme 1 – Knowledge: Certainty and Uncertainty. Additional data to support theme 1. (PDF 490 kb) [file 41927_2018_23_MOESM2_ESM.pdf]

Additional file 2. Participant data supporting Theme 1 - Knowledge: Certainty and Uncertainty

Structural model of progressive degeneration
• *"The cartilage is gone. It didn't all disappear at once, it's wearing out … you might be making something out of metal, if you beat on it enough, well it gets thinner... That's the way I look at my knee joint."*<br>*–James, 70 to 74 years old, knee pain 4 to 6 years*<br>• *"It's crapping out all the time, a little bit every year… a race against time."*<br>*–John, 65 to 69 years old, knee pain 4 to 6 years*<br>• *"You can hear it, you know, grinding. So to me it feels like bone-on-bone."*<br>*–Geoff, 60 to 64 years old, knee pain 14 to 16 years*<br>• *"I have quite bow knees. So obviously there are some structural changes happening."*<br>*–Linda, 50 to 54 years old, knee pain over 20 years*<br>• *"My leg muscles sort of work in reverse, you know, of trying to hold the joint apart."*<br>*–James, 70 to 74 years old, knee pain 4 to 6 years*<br>• *"[When the pain changes] I hope I'm not breaking any more cartilage."*<br>*–William, 60 to 64 years old, knee pain 4 to 6 years*
**Approaches to osteoarthritis management**
• *"It was a bit of a shock seeing [in the X-ray] just what little there is there... it woke me up and said 'right, well I can't continue fully on it. I've got to be aware of it all the time'."*<br>*–Anne, 60 to 64 years old, knee pain 10 to 12 years*<br>• *"Whatever I'm doing, it makes me think about really can I accomplish what I want to do. So you know, just to go for a walk, how far do I want to go and is there anywhere to rest?"*<br>*–James, 70 to 74 years old, knee pain 4 to 6 years*<br>• *"It makes me a bit lazier… you do things to protect your knee from what you think might be going to hurt."*<br>*–Karen, 60 to 64 years old, knee pain 18 to 20 years*<br>• *"[Changes in pain mean] your knee is reminding you to be careful and to watch out for what you do."*<br>*– Linda, 50 to 54 years old, knee pain over 20 years*<br>• *"I like playing golf so I bought myself a scooter to go round the golf course. To save walking, to save wear and tear on that right knee."*<br>*–George, 80 to 84 years old, knee pain 8 to 10 years*<br>• *"I wear a brace all the time. Give it a bit of support."*<br>*–Geoff, 60 to 64 years old, knee pain 14 to 16 years*<br>• *"They mightn't have done a great deal for me, but we're all at different stages and levels and all that, and it might help them."*<br>*–James, 70 to 74 years old, knee pain 4 to 6 years*<br>• *"You can't put that stuff [cartilage] back in there and so you just need to replace the joint at some stage."*<br>*–Karen, 60 to 64 years old, knee pain 18 to 20 years*

From: Darlow B, Brown M, Thompson B, Hudson B, Grainger R, McKinlay E, Abbott JH (2018) Living with osteoarthritis is a balancing act: An exploration of patients' beliefs about knee pain.
