# Supplementary material for: Living with osteoarthritis is a balancing act: an exploration of patients’ beliefs about knee pain
Source: BMC Rheumatol. 2018 Jun 12;2:15. doi: 10.1186/s41927-018-0023-x (PMC6390552; doi:10.1186/s41927-018-0023-x)
Supplement: Supplementary file 3 — Participant data supporting Theme 2 – Living with osteoarthritis. Additional data to support theme 2. (PDF 485 kb) [file 41927_2018_23_MOESM3_ESM.pdf]

Additional file 3. Participant data supporting Theme 2 - Living with osteoarthritis

The big picture
• *"It's not about keeping well in my knee or keeping well in my heart. It's just keeping well in my whole body. And there's no point in looking at one part, per se."*<br>*– John, 65 to 69 years old, knee pain 4 to 6 years*<br>• *"[That] I can still be active. That I can still enjoy my life…Yeah, I'm still 'Me'.*<br>*–Susan, 60 to 64 years old, knee pain 1 to 2 years*<br>• *"The swimming and the biking, I'd still do anyway, even if I didn't have a bung knee."*<br>*–Susan, 60 to 64 years old, knee pain 1 to 2 years*
**Living with osteoarthritis is a balancing act**
• *"You know it [exercise] should be helping because it should be strengthening the muscles above the knee. But you'll often find that after you've used it, the resting pain level is quite a lot higher."*<br>*–Karen, 60 to 64 years old, knee pain 18 to 20 years*<br>• *"I know there's not much cartilage there, which is the worrying thing really. And I don't want to wear it all out. But keeping your tendons and your muscles still toned up and working, surely that's got to be of benefit?"*<br>*–Anne, 60 to 64 years old, knee pain 10 to 12 years*<br>• *"It's that balancing act between the lawns have to get done and it's a cost I can't really meet to pay somebody else."*<br>*–Susan, 60 to 64 years old, knee pain 1 to 2 years*<br>• *"There's a fine line between… 'if there's no pain there's no gain' and it's too much pain and I don't want to do it."*<br>*–James, 70 to 74 years old, knee pain 4 to 6 years*<br>• *"I've got kind of like a limited range of pain medications that I'm prepared to take, so yeah you wind up walking along a bloody fine line."*<br>*–Mary, 70 to 74 years old, knee pain 14 to 16 years.*<br>• *"I don't want it to dominate my life, but sometimes it does."*<br>*–Brenda, 55 to 59 years old, knee pain 0 to 6 months*

From: Darlow B, Brown M, Thompson B, Hudson B, Grainger R, McKinlay E, Abbott JH (2018) Living with osteoarthritis is a balancing act: An exploration of patients' beliefs about knee pain.
